# Effectiveness of automated locomotor training in patients with acute incomplete spinal cord injury: A randomized controlled multicenter trial

**DOI:** 10.1186/1471-2377-11-60

**Published:** 2011-05-27

**Authors:** Markus Wirz, Carolien Bastiaenen, Rob de Bie, Volker Dietz

**Affiliations:** 1 Spinal Cord Injury Center, Balgrist University Hospital, Zurich, Switzerland; 2 Department of Epidemiology, Centre for Evidence-Based Physiotherapy, Maastricht University, Maastricht, The Netherlands

**Keywords:** Spinal Cord Injuries, Walking, Exercise Therapy

## Abstract

**Background:**

A large proportion of patients with spinal cord injury (SCI) regain ambulatory function. However, during the first 3 months most of the patients are not able to walk unsupported. To enable ambulatory training at such an early stage the body weight is partially relieved and the leg movements are assisted by two therapists. A more recent approach is the application of robotic based assistance which allows for longer training duration. From motor learning science and studies including patients with stroke, it is known that training effects depend on the duration of the training. Longer trainings result in a better walking function. The aim of the present study is to evaluate if prolonged robot assisted walking training leads to a better walking outcome in patients with incomplete SCI and whether such training is feasible or has undesirable effects.

**Methods/Design:**

Patients from multiple sites with a subacute incomplete SCI and who are not able to walk independently will be randomized to either standard training (3-5 sessions per week, session duration maximum 25 minutes) or an intensive training (3-5 sessions per week, session duration minimum 50 minutes). After 8 weeks of training and 4 months later the walking ability, the occurrence of adverse events and the perceived rate of exertion as well as the patients' impression of change will be compared between groups.

**Trial registration:**

This study is registered at clinicaltrials.gov, identifier: NCT01147185.

## Background

The general prognosis for regaining ambulatory function after a traumatic SCI ranges from 3% in initially complete SCI patients (according to the Standard Classification of the American Spinal Injury Association ASIA A [1]) to 95% in very incomplete lesions (ASIA D) [2]. It is reported that for patients with a motor complete and sensory incomplete SCI (ASIA B) the chance to become ambulatory is 50%. Those subjects with preserved algesia seem to recover to about the same extent as motor incomplete SCI subjects [3].

**Figure 1:**
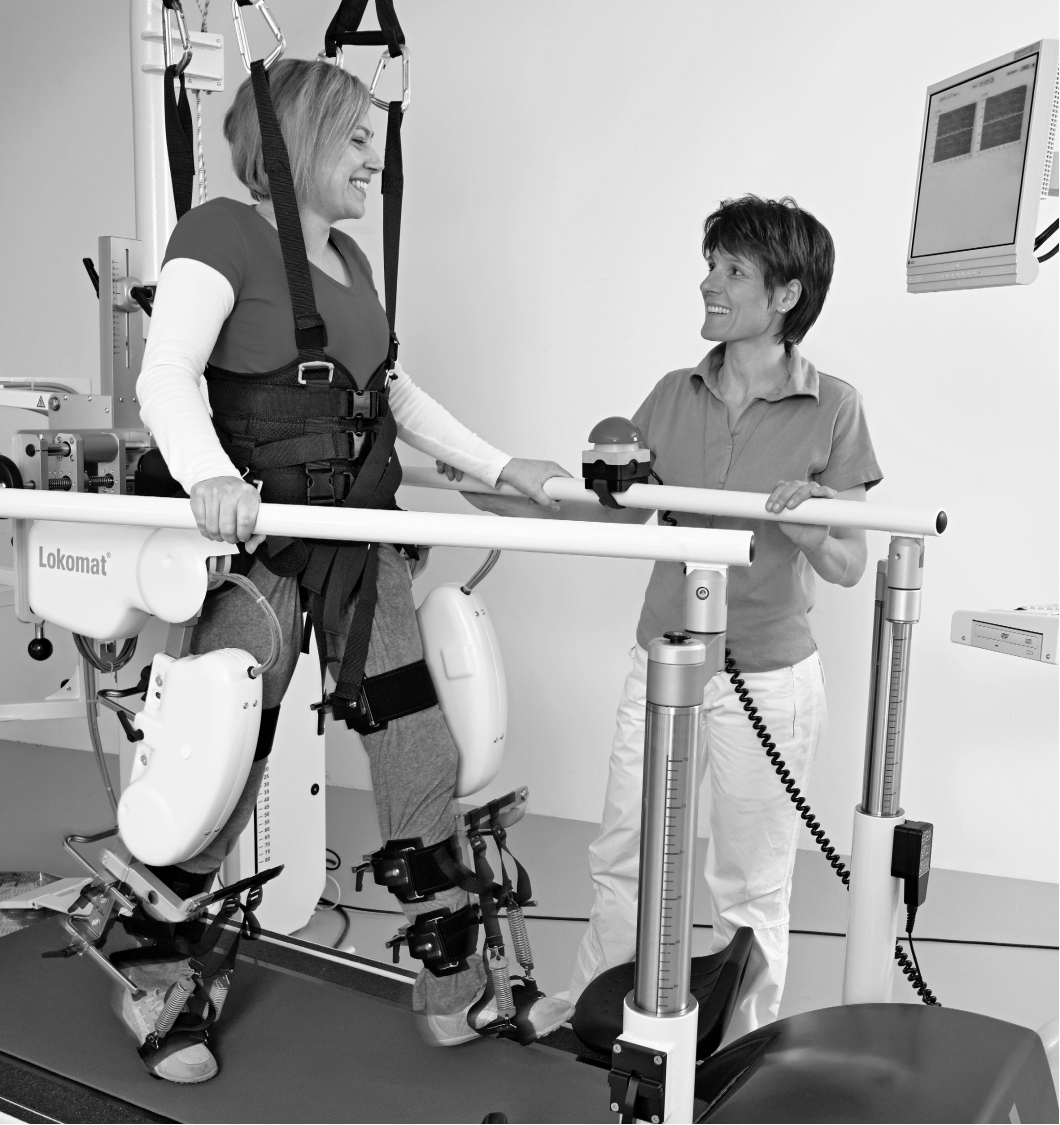
**The Lokomat is an actuated robotic exoskeleton for the training of ambulatory function on a moving treadmill**. The patient is secured by an overhead suspension system which partially reliefs the body weight. Position sensors and force transducers are used to monitor the efforts of the patient (Picture by courtesy of Hocoma AG).

The basis for locomotor training after acute spinal cord injury (SCI) in humans is provided by animal experiments which showed training induced plasticity of spinal locomotor centers [4]. In humans with a clinically complete spinal injury walking-like EMG activity can be elicited when subjects perform bodyweight supported and assisted stepping on a moving treadmill [5]. In SCI rehabilitation body weight supported treadmill training (BWSTT) has become established within the last 2 decades. In severely affected SCI subjects movements of the patient legs have to be manually assisted by two therapists. In some cases a third therapist might be needed to stabilize the pelvis. The shortcoming of such manual assisted BWSTT is that it is very exhaustive for the therapists and hence only allows for limited training time. In addition the application of movement assistance requires extra skills to maintain coordination between the two legs. In order to provide longer training sessions with a consistent movement pattern robotic gait devices were developed. These driven gait orthoses (DGO) become successively more established to treat individuals with a locomotor dysfunction such as incomplete SCI, stroke or traumatic brain injury [6]. A widely used DGO is the Lokomat (Hocoma AG, Volketswil, Switzerland, Figure 1) [7]. It has been shown that locomotor training with the Lokomat is feasible and that patients with a chronic incomplete SCI could improve gait speed and endurance as a response to an intensive training which lasted 8 weeks [8].

Patients with mild para- or tetraparesis become ambulatory within days or weeks without being trained using special forms of therapy e.g. BWSTT or Lokomat. In contrast, SCI subjects presenting with an incomplete but severe spinal paresis (i.e. ASIA B and C) are referred to an intensive and specific locomotor training by the Lokomat. So far this algorithm is based on expert consensus [9].

The strategy of rehabilitation after SCI or stroke is based on the principles of motor learning. Important characteristics of exercises have been identified. Beside task-specificity, task-variability, feedback or contextual interference the amount of exercise seems to be a key element [10,11]. The improvement of motor performance within rehabilitation may be due to true recovery or compensation [12]. Nevertheless, the above mentioned principles are valid regardless the underlying mechanism [11].

A meta analysis including studies of patients with stroke showed that augmented exercise therapy had a favorable effect on activities of daily living [13] and longer training duration was correlated with improved walking performance (positive dose-response relationship) [14]. One of the advantages of robotic devices like the Lokomat is the ability to prolong time for locomotor training compared to manually assisted training [15]. Yet it is not clear whether longer training duration results in an improved outcome or if certain endpoints in terms of walking capacity can be achieved within a shorter period of time.

The aim of the present study is therefore to evaluate whether SCI patients with severe sensory-motor deficit after acute traumatic SCI (ASIA B and C) profit from prolonged Lokomat training compared to patients who undergo the usual training paradigm. The hypothesis is that patients with a severe but incomplete SCI who undergo a prolonged Lokomat training achieve higher grades of walking ability compared to their counterparts who complete the training as suggested by experts.

The secondary aim is to evaluate how feasible prolonged locomotor training time is, i.e. whether there is an association between training intensity and adverse events e.g. increased spasticity or pain.

## Methods/Design

The design of the study was developed in accordance with the Consolidated Standards of Reporting Trials (CONSORT statement)[16].

This study will take place at multiple sites (i.e. Zurich/CH (leading center), Barcelona/E, Toledo/E, Heidelberg/D, Murnau/D, Nijmegen/NL and Glasgow/GBR). Local Ethics Committees at each center have approved (Barcelona, Toledo and Zurich) the study. Informed consent will be obtained from all subjects prior to participation. Patients will be included as they are referred to one of the participating centers (consecutive sample).

### Subjects

Patients with a subacute traumatic SCI initially categorized as ASIA B or C with a motor level between C4 and T12 and who are only partially able to walk (Walking Index for Spinal Cord Injury-WISCI ≤5 [17]) will be eligible. Subjects should be able to start the training within 60 days after trauma. Patients who do not comply to the requirements of the Lokomat training device (i.e. bodyweight >130 kg, body height >200 cm, leg length diff >2 cm, osteoporosis, instable fracture of lower extremity, restricted range of motion, decubitus ulcer of lower extremity) or with concomitant injury limiting walking ability (e.g. lower extremity fractures, instable spine fractures, Joint instability preventing weight-bearing, severe soft tissue lesion, traumatic brain injury) or with pre-existing medical conditions interfering with unrestricted walking (e.g. total joint replacement, chronic pain, osteoarthritis, polyneuropathy, cardiopulmonary disease) or who are older than 60 years or younger than 18 years will be excluded from participation. Patients who already participate in other rehabilitation or pharmacological study will also not be considered for participation.

In previous studies with stroke subjects [18-23] the mean difference in walking speed amounted to 0.0418 m/s. This value was used to calculate the sample size further assuming a standard deviation of 0.05 m/s, a statistical power of 0.8 and a significance level of 0.05. The calculation resulted in the requirement of 23 subjects in each group to be able to reject the null hypothesis.

### Randomization

Patients will be randomly assigned to either the intervention or the control group using a computer generated 4-block randomization scheme. The allocation will be performed by an independent person not otherwise involved in the study. The responsible researchers at each center request the group allocation by mail.

### Intervention

The locomotor training with the Lokomat device should start within approximately 30 days but not later than 60 days after the SCI. The observation period of the training for this study lasts 8 weeks. For the initial 5 trainings there are no defined specifications. These trainings serve to optimize the setup and for the patients to familiarize with the robotic locomotor training device. During subsequent trainings following guideline will be adopted to adjust the training to the actual capacity: body weight unloading will be reduced to the least tolerated amount (no knee buckling or toe dragging). The walking speed will be set within the range of 1.6 to 3.1 km/h and the guidance force in the range from 100% (full assistance) to minimum tolerated. The training session will be shaped in the following way: 3 min walking without specification (warm-up period) after that, every 3^rd ^minute either speed, visual feedback or guidance force will be changed. This will avoid that the training becomes monotone and lacks challenge.

Patients who will be assigned to the intervention group receive one or two Lokomat trainings per day on 3-5 days per week. The Lokomat walking time per day should not be shorter than 50 min.

### Control

Patients of the control group receive one Lokomat training per day on 3-5 days per week. The Lokomat walking time per day should not be longer than 25 min.

### Outcome

Outcome data will be assessed in the respective centers by therapists and medical doctors. Hereafter the data will be sent anonymously to the PI for analyses. Due to the characteristic of this study neither the therapists nor the assessors nor the patients can reliably be blinded regarding the group allocation. The principal investigator (PI) who is the first author of this report (MW) and will be involved in the analyses will not be aware of the respective intervention.

In order to describe the characteristics of the included subjects, demographic and clinical data will be assessed. The primary outcome is the self selected walking speed using the Ten Meter Walking Test (TMWT) [24]. It will be assessed at baseline, bi-weekly during training, and at the six months follow-up. In addition the following items will be assessed at baseline, bi-weekly during training, and after six months: the Walking index for spinal cord injury -WISCI [17] (an ordinal scaled index for the assessment of walking capabilities with 21 categories. Zero represents that the patient is not able to stand or walk, the maximum of 20 means that the patient can walk without bracing, walking aids or personal assistance), the maximum walking speed, the ASIA classification [1], the detailed Spinal Cord Independence Measure-SCIM [25], the modified Ashworth scale-MAS [26] of hip and knee joints, and the Penn spasm frequency scale [27]. During the training period the mechanical stiffness and the maximum voluntary torque of the legs as well as the cooperation of the patient during the training will be assessed bi-weekly using the force transducers of the robotic training device. For every training the distance walked, walking speed, walking time and body weight unloading, the rate of perceived exertion as well as the occurrence of any events (e.g. skin breakdown or joint stress or scheduling problems) will be assessed. At the end of the training period, i.e. after 8 weeks the patients subjective impression about the success of the training will be assessed using the Patients' Global Impression of Change Scale-PGIC [28].

Since at the time of the study all patients will undergo a rehabilitation program, they receive along with the Lokomat training the usual rehabilitative therapy (i.e. physio and occupational therapy). In order to assure that the standard rehabilitation program is comparable between the participating centers, therapy schedules of one week will be collected of four patients per center. These schedules should contain the amount of therapy and roughly the content of the therapies (e.g. focus on arm or leg or standing and walking or other activities).

### Analyses

All analyses will be performed in the leading center by the PI. He receives the data in form of an electronically completed case record form after completion of the training. For the analyses, the data will then be incorporated into the statistical software (PASW statistics 17.0, IBM-SPSS Inc. Chicago/IL).

In order to describe the characteristics of the sample, descriptive statistics will be applied. For the analysis of training induced differences between intervention and control group, T-Test for independent groups or the non-parametric correspondent depending on the outcome variable will be applied. In addition, multilevel models will be used to evaluate potential confounding factors (e.g. center effects, age differences, neurological level of lesion or ASIA) as required.

## Competing interests

The authors declare that they have no competing interests.

## Authors' contributions

MW developed the design of the study and acts as the PI. CB, RdB and VD were all involved in the development of the design. All authors read and approved the final manuscript.

## Pre-publication history

The pre-publication history for this paper can be accessed here:

http://www.biomedcentral.com/1471-2377/11/60/prepub
